# Supplementary material for: Patterns of Intron Gain and Loss in Fungi
Source: PLoS Biol. 2004 Nov 30;2(12):e422. doi: 10.1371/journal.pbio.0020422 (PMC532390; doi:10.1371/journal.pbio.0020422)
Supplement: Table S1 — Also available at http://genes.mit.edu/NielsenEtAl/. (4.3 MB ZIP). [file pbio.0020422.st001.zip › NielsenEtAl/html/952.html]

AN7832.1.NCU04938.1.MG00779.1.FG11008.1


```
 CLUSTAL W (1.82) Multiple Sequence Alignments - Introns Inserted


Sequence 1: AN7832.1	648 aa
Sequence 2: FG11008.1	641 aa
Sequence 3: MG00779.1	646 aa
Sequence 4: NCU04938.1	671 aa
Alignment Length: 676 aa
Number Identitical Residues: 296 aa
Alignment Score (without introns) 15428


MG00779.1 	MKLPQLFGQLCLSFTLAAAVDLTGYEYIVVGSGAGGGPLAARLAMAGKKTLLLEAGDDQG
NCU04938.1	MIFHHLLGLALAATPLALAVELTGYEYIVVGSGAGGGPLAARLAMAGHKTLLIEAGNDQG
FG11008.1 	-MRQSFLTGLLAAPLSAYAVALTGYEYIVVGSGAGGGPLAARLAMAGHKTLLIEAGDDYA
AN7832.1  	MMLPRLFSFLAATAGVARAVNITGYEYVVVGSGAGGGPLAARLALAGHKTLLLEAGDDQG
          	     ::     :   * ** :*****:****************:**:****:***:* .

MG00779.1 	ANLNYTVPAYHSRSSEDDSMSWDFFVRNYADEAQQARNFKTTYETPDGGEYTGLNPPPGS
NCU04938.1	NNVNVTVPAYSTRATEDEKLSWAFYVRHYPDDEQQARDWKTSYRLPNGTIYTGLSPPEGA
FG11008.1 	TT-NYTVPAYSANASEDPEISWNFFVKHYADDERQARDFKTTYDTPDGE-YTGLNPPADA
AN7832.1  	QTYNYSIPAYSARASEDEKLAWNFFVHHYADDERQARDFKTSYETPSGEIYTGLNPPEGS
          	 . * ::*** :.::** .::* *:*::*.*: :***::**:*  *.*  ****.** .:

MG00779.1 	KMKGVLYPRTG~TLGGCTAHNAMVTIYPFRSDFDDLATLTGDKSWSATNMRKYFAKMEDN
NCU04938.1	EMLGTFYPRAG~TLGGCTAHNALLAVYPFQSDFQYIADLTGDDGWLPDNMRQHFIALEDK
FG11008.1 	KLKGTLYPRTQ~ALGGCTAHNALIAVYPHQSDFEYIATLTGDDSWSAKNMRKYFVKAEDN
AN7832.1  	TLLGTLYPRTG1TLGGCTAHNALIAVYPHQSDFEYISTLTGDSSWSPENMRGYFEKLERN
          	 : *.:***:  :*********::::**.:***: :: ****..* . *** :*   * :

MG00779.1 	HHLLPGKAGHGYSGWLSTQTAPLTIALGDPQLLGFFVGAAAALTNWSTNLVFNLATLFAG
NCU04938.1	NYPQGGYYGHGQDGWLSIELPTLTTP--DSELSSLVVGGAYALGNATD-SPISLTSLATN
FG11008.1 	NHLLPLQPGHGYDGWLSTETAPLSIPLGDAQLMSLLVGGATALSEVTG-ELLTMETLIAG
AN7832.1  	KYLLPGLEGHGYDGWLQTETAPLSLVLKDPQLLSMLTGGAFALGNWTD-NIYNIGTLLAG
          	::      *** .***. : ..*:    *.:* .:..*.* ** : :     .: :* :.

MG00779.1 	DANEDSALRDSRPGNFQVPLSTRDGARISSRDFIVAVRDAKNPDGSKMYPLDVRTDALVT
NCU04938.1	DLNADTIARDHDPSYNGIPISTRNGTRNGSRDFVVAVRDALNPDGSKKYPLDVRLNCFVT
FG11008.1 	DANADTLARDQKPGYYQIPLSTKSASRVGPREFILSVRDAKFDNGTKRYPLDVRTNAYVT
AN7832.1  	DANADRTTRDTVPGYYQIPVSTDDAHRNGAREFIIAVRDAKNADGSKKYPLDVRMNTHVT
          	* * *   **  *.   :*:** .. * ..*:*:::****   :*:* ****** :  **

MG00779.1 	KVLFDNS----TTPPRTTGVEFRDGKYLYRASRRSKNAGQG-------------VRGTAT
NCU04938.1	KITFDDTDWDETGEPVATGVEFLDGEYLYRASPLSSSSSSSSKHAKHGGKHSNGTPGSAK
FG11008.1 	KVLFDES----EKTPRATGVEFLDGKHLYSASPLS-TGAAG-------------VAGNAT
AN7832.1  	KVTFDET----QDPPRAAGVEFLDGKHLYKASPLSNTASAG-------------IPGAAT
          	*: **::       * ::**** **::** **  *.... .               * *.

MG00779.1 	ASREVILAAGAYNSPQILKLSGIGPAAELQRFDIPVVKDLPGVGANLQDHYEVSVQGTTP
NCU04938.1	ASREVIISGGTYNSPQILKLSGIGPQDELTKFGIPVVVDLPGVGGNLQDHYEISVNVKVP
FG11008.1 	ASREVIVAGGVYNTPQILKLSGIGPADELKKFDIPVVVDLPGVGTNLQDHYEISVQGNTP
AN7832.1  	ASREVIVAGGVYNSPQILKLSGIGPADELEKFGIKVIKDLPGVGTNLQDHYEISVQGRIE
          	******::.*.**:***********  ** :*.* *: ****** *******:**:    

MG00779.1 	SDFSSLKGCTFSPET-GEDPCLTRWQNPILGNRGIYSSSGFATAIYYKSKEAKGSDWDVF
NCU04938.1	QDWTVLENCTFLLFDNTTDPCLDRWENPVMGNRGTYASNGVPVVMLYKSSAAEGDDWDLF
FG11008.1 	ENFTAFDGCTFSLYT-DEDPCLDRWRQPVLGDHGIYSSSGLAATMFYKSTTAEQNNFDIF
AN7832.1  	NDFSCLDGCTFGIRG-EEDPCIDRWESPILGDRGIYSSPGLAATMYYKSSVTADNSFDIF
          	.::: :..***       ***: **..*::*::* *:* *....: ***. :  ..:*:*

MG00779.1 	SFGGPANFRGYFPGYSVNATARHNVFSWAVLKGHPRNAAGSVTLRSADPLDVPDIVYDYF
NCU04938.1	TFGGPLNFAGYYPGYSVDLVAEHNWWTWAILKAHPRNRAGTVTLRSSDPLDTPEINFNYF
FG11008.1 	AFGGPVNFRGYFPKYAVNATIDHDFFSWAILKAHPRNTAGNVLLRSADPLDTPAITFNYF
AN7832.1  	CFGGPVNFRGYFPDYSINATDEHNWFTWAILKAHPRNTAGTVTLQSSNPLDVPKITFNYF
          	 **** ** **:* *::: .  *: ::**:**.**** **.* *:*::***.* * ::**

MG00779.1 	KDAG--SDKDLRAMTEGIKLFRTALKLQLLGQPLREELPGDAVQTDEQIADYIRDTAWGH
NCU04938.1	EYGSGDYEADLTAMREGICLARDALRRSGVC--TQEVRPGADIQSDEDLDQYIIDDAWGH
FG11008.1 	DTGNGDSDKDLQAMYEAVELARKAFASQPVK--TQETLPGDHVKTQEDVETYIKDNTWGH
AN7832.1  	DTGVGDYDADLTALYEAVELARDAFHRQPIN--VTEVLPGAAVTSKEDIETYVKDSAWGH
          	. . .. : ** *: *.: * * *:  . :     *  **  : :.*::  *: * :***

MG00779.1 	HASSTCAIGVDGDSMAVLDSSFRVRGVTGLRVADASVYPKIPGTFTAVSTYMIGEKAADV
NCU04938.1	HASSTCPIGPDGDPMAVLDSKFRVRGVKGLRVVDASVYPRIPGTFTQISTYMVAEKAACN
FG11008.1 	HASSTCPIGADDDKMAVLDSKFRVRGVAGLRVVDASVYPRIPGTFTAVSTYMVAEKAADI
AN7832.1  	HASCTCPIGADDDPMAVLDSKFRVRGVSGLRVVDASVYPKIPGTFTAVSTYMVAEKAADD
          	***.**.** *.* ******.****** ****.******:****** :****:.****  

MG00779.1 	ILSQESA----------
NCU04938.1	ILEGLENEEVDDGYWRV
FG11008.1 	MLAELEE----------
AN7832.1  	ILTELAAASS-------
          	:*      .
```
